# Vital staining as a tobacco cessation aid and diagnostic adjunct: a cross-sectional study on high-risk Indian patients

**DOI:** 10.1038/s41405-025-00382-0

**Published:** 2025-11-28

**Authors:** Satya Ranjan Misra, Abhijeet Satpathy, Rupsa Das, Krishna Madhuri Dash

**Affiliations:** Department of Oral Medicine & Radiology, Institute of Dental Sciences, Siksha ‘O’ Anusandhan University, Bhubaneswar, Odisha India

**Keywords:** Oral cancer detection, Dentistry

## Abstract

**Introduction:**

Oral squamous cell carcinoma (OSCC) ranks among the most prevalent cancers worldwide, typically arising from oral potentially malignant disorders (OPMDs). Early detection in high-risk individuals is critical for improving patient outcomes. This cross-sectional study assessed the diagnostic accuracy of sequential vital staining with 5% Methylene Blue (MB) and 3% Lugol’s Iodine (LI) and explored its influence as a visual behavioural motivator to encourage tobacco cessation among Indian tobacco users.

**Methods:**

Fifty adult tobacco users presenting with oral mucosal lesions underwent in vivo double vital staining. Each lesion was first stained with MB, followed by LI. Clinically retained MB and non-retained (negative) LI areas were recorded. Targeted biopsies were obtained from stained and unstained sites. Histopathological findings were correlated with staining patterns to determine sensitivity, specificity, and overall diagnostic accuracy.

**Results:**

MB staining alone demonstrated high diagnostic yield, with sensitivity of 94.7%, specificity of 87.1%, and overall accuracy of 90.0%. Combining MB and LI increased sensitivity to 100%, ensuring no dysplastic lesions were missed, but reduced specificity to 67.7%, leading to more false positives. Clinically, the vivid contrast provided by double staining enhanced lesion delineation, and many participants reported that seeing the stained lesions heightened their awareness and spurred discussions on tobacco cessation.

**Conclusion:**

The MB-LI sequential staining technique offers exceptional sensitivity for identifying dysplasia in tobacco-related oral mucosal lesions. Its bright visual feedback not only supports early diagnosis but also appears to function as a powerful motivational tool, prompting self-reflection and encouraging cessation behaviour among tobacco users. These findings suggest that vital staining has dual utility: as an effective diagnostic adjunct and as a behavioural intervention aligned with tobacco-cessation strategies recommended by dental professionals.

## Introduction

Oral squamous cell carcinoma (OSCC) remains a significant global health challenge, closely tied to tobacco use, especially in South Asia, where both smoked and smokeless forms are deeply entrenched. In India alone, approximately 113,300 new cases are reported annually, with age-standardized rates around 20 per 100,000 people, accounting for nearly 30% of all cancers nationally [1]. Despite the visibility of the oral cavity, early lesions are often overlooked until advanced stages due to a lack of symptoms, low awareness, and limited access to routine screening [2]. Oral squamous cell carcinoma (OSCC), which frequently develops from oral potentially malignant disorders (OPMDs) such as leukoplakia, erythroplakia, and oral submucous fibrosis, constitutes most cases [3, 4]. Traditional screening using conventional oral examination techniques may miss subtle early dysplasia [5]. Hence, accessible, cost-effective, user-friendly diagnostic adjuncts are urgently needed.

Vital staining using dyes such as toluidine blue (TB), methylene blue (MB), and Lugol’s iodine (LI) offers a non-invasive, chairside method to highlight dysplastic mucosa and guide biopsy [6]. Although TB’s clinical utility is well-documented, yet its mucosal toxicity can limit frequent application [6]. In contrast, MB binds selectively to DNA-rich dysplastic tissue and has a favourable safety profile, making it a valuable alternative [7]. The target of LI is glycogen, staining normal cells brown and leaving dysplastic or malignant areas unstained due to glycogen depletion helps delineating the normal from the dysplastic area [8, 9].

Few studies have found that combining MB and LI enhances diagnostic performance. For instance, double staining has delineated lesion borders more accurately, demonstrating promising results in esophageal and oral mucosal lesions [9, 10]. Moreover, Indian clinical trials indicate that MB achieves sensitivity and specificity of approximately 89–90% and 62–66% respectively, while LI ranges between 83–87% and 50–84% [7, 10]. Beyond diagnostics, vital staining may play a powerful psychological role. The visual impact of stained lesions often prompts patients to reflect on their habits, initiating tobacco-cessation conversations in real time. In many cases, individuals expressed concern upon seeing stained mucosa and asked for advice on quitting, suggesting a potential “behavioural catalyst” effect [11].

The objectives of this cross-sectional study are:

1. Compare diagnostic performance of MB alone versus sequential MB–LI double staining in detecting dysplasia among high-risk tobacco users in India [11, 12].
2. Evaluate patients’ responses to visual staining and its potential to serve as a motivational trigger during routine oral screening [6, 13, 14].

By examining both diagnostic accuracy and psychological impact, this study explores dual utility of vital staining as a diagnostic adjunct and as a catalyst for tobacco cessation among high-risk populations [15, 16].

## Materials and Methods

### Study design and ethical approval

This was a cross-sectional observational study conducted at a tertiary dental care teaching hospital in India. Ethical clearance was obtained from the Institutional Ethics Committee (Ref. No. IEC/2023/04/021), and all procedures were carried out in accordance with the principles of the Declaration of Helsinki (2013 revision) [17]. Written informed consent was obtained from all participants prior to enrolment.

### Patient selection

Fifty adult patients (aged ≥18 years) with a documented history of tobacco use and presenting with clinically evident oral mucosal lesions were recruited. Patients were excluded if they had systemic contraindications to biopsy, a known hypersensitivity to methylene blue (MB) or Lugol’s iodine (LI), or a history of treatment for oral potentially malignant disorders (OPMDs) or malignancy [4].

### Clinical examination and staining protocol

Detailed case histories were obtained, including patient demographics and behavioural habits such as type, frequency, and duration of tobacco (chewed or smoked) and alcohol use. A thorough intraoral examination was performed to evaluate lesion morphology, site, and clinical staging [17].

The staining procedure consisted of a sequential dual-application method [18]. Initially, participants rinsed with 1% lactic acid for 30 s to eliminate surface debris. A sterile cotton applicator was then used to apply 5% MB directly to the lesion. After 30 s of contact time, excess dye was removed by a second lactic acid rinse. This was followed by the topical application of 3% LI to the same area and adjacent mucosa. A final rinse was performed before visual assessment of dye uptake. Staining outcomes were categorized as follows:

**Table:** 

Staining outcome	Interpretation
Deep royal blue coloration	**MB-positive**
No staining or faint bluish hue	**MB-negative**
Dark brown coloration (suggestive of glycogen-rich normal mucosa)	**LI-positive**
Pale yellow/ unstained regions (indicating dysplastic/ malignant changes)	**LI-negative**

**Fig. 1:**
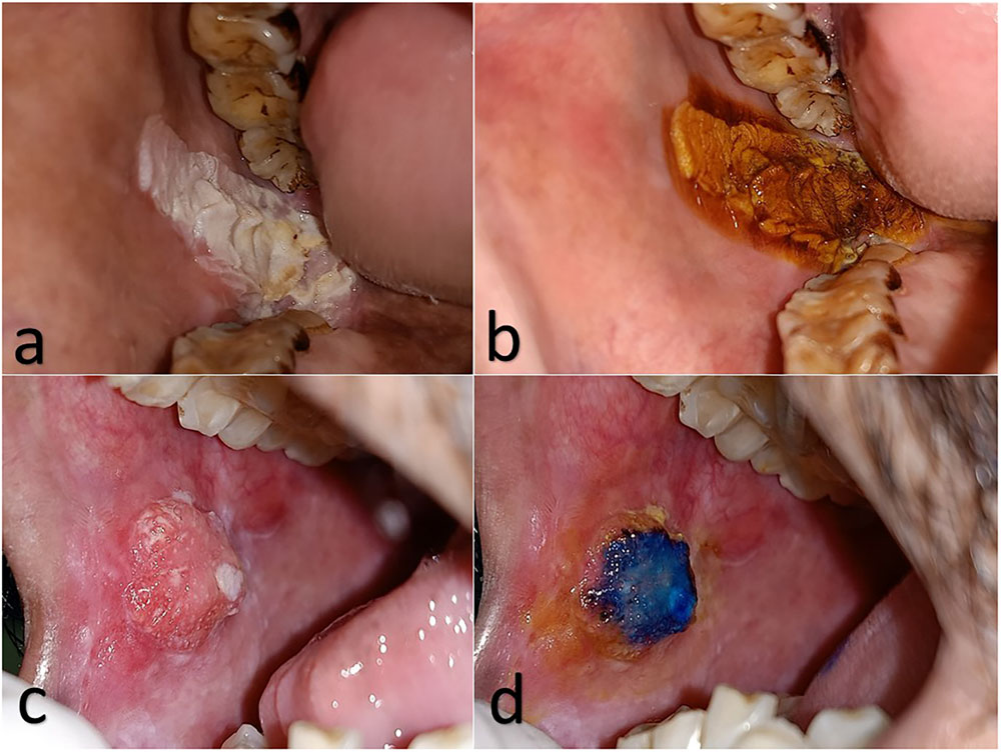
Dual staining of tobacco induced oral lesions. shows sequential staining using MB and LI on a homogenous leukoplakic patch (**a**) and a malignant ulceroproliferative growth (**c**) and their appearance post staining. The leukoplakic patch stains brown (**b**) and does not retain any blue staining while the border of the malignant growth is demarcated by the brown LI stain and the central dysplastic area stains deep royal blue colour indicating epithelial dysplasia (**b**).

A representative picture of both staining outcomes is shown in Fig. 1.

### Biopsy and histopathological correlation

Oral mucosal sites exhibiting MB positivity and/or LI negativity were selected for incisional biopsy under local anaesthesia. Biopsy specimens were processed and reviewed by a blinded oral pathologist. The presence or absence of epithelial dysplasia was recorded.

### Assessment of behavioural impact

Following the vital staining procedure and explanation of the results, participants’ readiness to quit tobacco was assessed using the Contemplation Ladder [19–22]. The Ladder is a visual analogue scale ranging from 0 to 10, where 0 represents “No thought of quitting” and 10 represents “Taking action to quit”. Participants were asked to select the number that best represented their current readiness to quit tobacco after having seen the stained lesions. Additionally, a single-item question (“How ready are you to quit tobacco after seeing the results of this stain?”) was administered on a scale of 1 (not at all ready) to 10 (very ready). Verbal feedback regarding their perception of the staining was also recorded qualitatively.

### Statistical analysis

Diagnostic performance parameters, including sensitivity, specificity, positive predictive value (PPV), negative predictive value (NPV), and overall accuracy, were calculated for MB staining alone and for the combined MB–LI protocol [13]. Analyses were conducted using 2×2 contingency tables, with histopathological diagnosis as the reference standard [13].

### Demographic profile of cases- age and gender distribution

Males are predominant among the study samples with a share of 41(82.0%), and female 9(18.0%). The overall mean ± SD and median (IQR) of age were 49 ± 14 years and 48(38–61) years, respectively. The mean ± SD and median (IQR) of age for males were 48 ± 13 years & 48 (41–59) years, and that for females were 51 ± 18 years & 47 (38-64) years. (Table 1).

**Table 1:** Descriptive Statistics of Age by gender.

Age	Gender		
	Male	Female	Total
Count	41	9	50
Row N %	82.0%	18.0%	100.0%
Mean	48	51	49
Standard Deviation	13	18	14
Minimum	25	25	25
Maximum	79	79	79
Range	54	54	54
Median	48	47	48
Q1 (1^ST^ Quartile)	41	38	38
Q3 (3^rd^ Quartile)	59	64	61

Tobacco used by the sample subjects: Out of 50 sample subjects, 35 (70.0%) were chewing tobacco, 9 (18%) smoking tobacco habits and 12% both chewing and smoking tobacco. (Table 2).

**Table 2:** Type of Tobacco use by the individuals.

Type of Tobacco use	Frequency	Percent	Cumulative Percent
Chewing	35	70.0	70.0
Smoking	9	18.0	88.0
Chewing & smoking	6	12.0	100.0
Total	50	100.0	

### Chewing frequency and duration

Out of 50 cases, 9 did not chew any tobacco. The minimum chewing frequency is 2 per day and a maximum of 45 per day. The average frequency was 15 ± 11 units per day. The median frequency with Inter Quartile Range (IQR) of daily chewing is 10 (7–20). The mean chewing and median chewing for males were 14 and 10, and for females, were 13 and 12. Even though the mean and median frequency of chewing for females were higher than that of males, the difference was not statistically significant (*p* = 0.430).

The minimum chewing duration is 1 year, and the maximum is 25 years. The average duration of chewing was 10 ± 5 years. The median duration of chewing with the Inter Quartile Range (IQR) was 10 (6–12). The mean chewing and median chewing duration for males were 11 and 10, and for females, were 10 and 10 years. The difference was not statistically significant (*p* = 0.055). The details are shown in the Table 3.

**Table 3:** Descriptive statistics of Chewing frequency and duration.

Chewing habit		Gender		
		Male	Female	Total
Chewing frequency (unit/day)	Missing	9	0	9
	Mean	14	17	15
	Standard Deviation	10	13	11
	Minimum	2	2	2
	Maximum	40	45	45
	Median	10	12	10
	Percentile 25	7	10	7
	Percentile 75	20	20	20
Chewing duration (Years)	Missing	10	0	10
	Mean	11	10	10
	Standard Deviation	6	3	5
	Minimum	1	4	1
	Maximum	25	12	25
	Median	10	10	10
	Percentile 25	5	10	6
	Percentile 75	15	12	12

Independent Sample test p value for comparison of mean Chewing frequency by gender 0.430 and mean Chewing duration by gender = 0.055.

### Smoking frequency and duration

Out of 50 cases, 35 did not smoke. All nine females did not smoke. The minimum frequency of smoking is 2 per day and a maximum of 40 per day. The average frequency was 15.6 ± 11.9 per day. The median frequency with Inter Quartile Range (IQR) of smoking per day is 14 (5–20). The minimum smoking duration is three years, and the maximum is 20 years. The average duration of smoking was 9.7 ± 6.3 years. The median duration of chewing with the Inter Quartile Range (IQR) was 8 (5–15). The details are shown in Table 4.

**Table 4:** Descriptive statistics of Smoking frequency and duration by gender.

		Gender		
		Male	Female	Total
Smoking frequency per day	Count	41	9	50
	Missing	26	9	35
	Mean	15.6	.	15.6
	Standard Deviation	11.9	.	11.9
	Median	14	.	14
	Percentile 25	5	.	5
	Percentile 75	20	.	20
	Minimum	2	.	2
	Maximum	40	.	40
Smoking duration	Count	41	9	50
	Missing	26	9	35
	Mean	9.7	.	9.7
	Standard Deviation	6.3	.	6.3
	Median	8	.	8
	Percentile 25	5	.	5
	Percentile 75	15	.	15
	Minimum	3	.	3
	Maximum	20	.	20

### Alcohol frequency and duration

Apart from the habit of tobacco, some have other habits like taking alcohol. Out of 50 sample subjects, eight were consuming alcohol, all of whom were males. Of these eight male patients, three consumed once a month, three-four times a month, and two five times a month. The minimum duration of alcohol consumption is one year and a maximum of 2 years. The average duration of alcohol was 1.1 ± 0.4 years. Table 5 depicts the details of alcohol consumption.

**Table 5:** Alcohol frequency and duration by gender.

			Gender		
			Male	Female	Total
Alcohol frequency per day	3	Count	3	0	3
		%	37.5	0.0	37.5
	4	Count	1	0	1
		%	12.5	0.0	12.5
	5	Count	2	0	2
		%	25.0	0.0	25.0
	10	Count	2	0	2
		%	25.0	0.0	25.0
Alcohol_duration (year)	Count		41	9	50
	Missing		33	9	42
	Mean		1.1	.	1.1
	Standard Deviation		0.4	.	0.4
	Minimum		1	.	1
	Maximum		2	.	2

### Clinical diagnosis by gender

The clinical diagnosis of sample patients is presented in Table 6. The most common diagnosis was 10 (20%) cases of oral submucous fibrosis, followed by 8 (16%) cases of Malignant Ulcer and 7 (14%) cases of Malignant ulcero-proliferative growth. These three diagnoses together constituted half of the cases. The other diagnoses were actinic cheilitis, chronic nonhealing ulcer, erythroleukoplakia, Verrucous Leukoplakia, Homogenous leukoplakia, non-homogenous leukoplakia, Oral lichenoid lesion, Oral lichen planus, tobacco-induced Lichenoid Reaction, tobacco pouch Keratosis, and verrucous carcinoma.

**Table 6:** Clinical Diagnosis by gender.

Clinical Diagnosis	Gender					
	Male		Female		Total	
	No.	%	No.	%	No.	%
Actinic cheilitis	1	2.4	0	0.0	1	2.0
Chronic non-healing ulcer	2	4.9	0	0.0	2	4.0
Erythroleukoplakia	1	2.4	0	0.0	1	2.0
Homogenous leukoplakia	4	9.8	1	11.1	5	10.0
Verrucous leukoplakia	1	2.4	0	0.0	1	2.0
Malignant Ulcer	4	9.8	4	44.4	8	16.0
Malignant growth	1	2.4	0	0.0	1	2.0
Malignant ulcero-proliferative growth	7	17.1	0	0.0	7	14.0
Non-homogenous leukoplakia	1	2.4	0	0.0	1	2.0
Oral Lichenoid Lesion	4	9.8	2	22.2	6	12.0
Oral Lichen Planus	1	2.4	2	22.2	3	6.0
Oral submucous fibrosis	10	24.4	0	0.0	10	20.0
Tobacco induced LR	1	2.4	0	0.0	1	2.0
Tobacco pouch Keratosis	2	4.9	0	0.0	2	4.0
Verrucous carcinoma	1	2.4	0	0.0	1	2.0
Total	41	100.0	9	100.0	50	100.0

Out of 9 cases of females, the most common diagnosis was 4 cases of Malignant Ulcer. This is followed by two instances of Oral lichenoid lesion and oral lichen planus each. Out of 41 cases of males, the most common diagnosis was 10 (20%) cases of oral submucous fibrosis, 7 (14%) cases of Malignant ulcero-proliferative growth, and 4 cases of Oral lichenoid lesion and malignant ulcers each.

### Histopathological diagnosis by gender

Histopathological diagnosis is the most confirmatory diagnosis, and the finding has been tabulated in Table 7. The most common diagnosis was well-differentiated squamous cell carcinoma (9, 18% cases), and Oral lichen planus (8, 16%). These are followed by Oral submucous fibrosis, oral lichenoid lesions, and Non-specific inflammation, with 6 (12%) cases each. Out of 9 cases of females, the most common diagnosis was 4 cases of oral submucous fibrosis. This is followed by two instances of the Oral lichenoid lesion and Oral lichen planus each. Out of 41 cases in males, the most common diagnosis was 8 (20%) cases of well-differentiated squamous cell carcinoma and 7 (17%) cases of oral lichen planus. This is followed by 4 cases of OLL and MU each.

**Table 7:** Histopathological diagnosis by gender.

Histopathological Diagnosis	Male		Female		Total	
	No.	%	No.	%	No.	%
Epithelial Dysplasia	3	7.3	1	11.1	4	8.0
Hyperkeratosis	3	7.3	0	0.0	3	6.0
Hyper-orthokeratosis	1	2.4	0	0.0	1	2.0
Hyperkeratosis with dysplasia	1	2.4	0	0.0	1	2.0
Hyperparakeratosis	1	2.4	0	0.0	1	2.0
Moderately differentiated Squamous cell Carcinoma	4	9.8	0	0.0	4	8.0
Nonspecific	0	0.0	0	0.0	0	0.0
Non-specific inflammatory	5	12.2	1	11.1	6	12.0
Lichenoid Reaction	5	12.2	1	11.1	6	12.0
Oral Lichen Planus	7	17.1	1	11.1	8	16.0
Oral Submucous Fibrosis	2	4.9	4	44.4	6	12.0
Papilloma	1	2.4	0	0.0	1	2.0
Well-differentiated squamous cell carcinoma	8	19.5	1	11.1	9	18.0
Total	41	100.0	9	100.0	50	100.0

### Diagnostic evaluation of Methylene blue staining

Tables 8 and 9 evaluate the Methylene Blue staining as a diagnostic adjunct in tobacco-induced oral mucosal lesions for epithelial dysplasia. The sensitivity was 94.74% with 95% CI (73.97% to 99.87%). The specificity was 87.10% with 95% CI (70.17% to 96.37%). The positive predictive value was found to be 81.82% with 95% CI (64.18% to 91.87%), negative predictive value was 96.43% with 95% CI (79.95% to 99.46% and the accuracy was 90.00% with 95% CI (78.19% to 96.67%).

**Table 8:** Methylene blue test and Epithelial dysplasia cross-tabulation.

		Epithelial dysplasia		Total
		Positive	Negative	
Methylene blue test	Positive	True positive = 18	False positive = 4	22
	Negative	False Negative = 1	True Negative = 27	28
Total		19	31	50

**Table 9:** Diagnostic Evaluation of Methylene blue staining versus Epithelial dysplasia.

Statistic	Value	95% CI
Sensitivity	94.74%	73.97% to 99.87%
Specificity	87.10%	70.17% to 96.37%
Positive Predictive Value (*)	81.82%	64.18% to 91.87%
Negative Predictive Value (*)	96.43%	79.95% to 99.46%
Accuracy (*)	90.00%	78.19% to 96.67%

(*) These values are dependent on disease prevalence.

The value of sensitivity, specificity, positive predictive value, and negative predictive value indicated that the methylene blue test, as a diagnostic adjunct in tobacco-induced oral mucosal lesions for detecting epithelial dysplasia, is very high.

### Diagnostic evaluation of double staining using Methylene blue and Lugol iodine in dysplastic oral mucosal lesions

Tables 10 and 11 present the evaluation of methylene blue & Lugol iodine as diagnostic adjuncts in tobacco-induced oral mucosal lesions to detect epithelial dysplasia. The sensitivity was 100% with 95% CI (82.35% to 100.00%). The specificity was 67.74% with 95% CI (48.63% to 83.32%). The positive predictive value was found to be 65.52% with 95% CI (53.29% to 75.99%), the negative predictive value was 100.00% with 95% CI (83.89% to 100.00%), and accuracy was 80.00% with 95% CI (66.28% to 89.97%). The sensitivity value is very high, while specificity is low, below 67%, when both methylene blue and Lugol iodine were combined to detect epithelial dysplasia in tobacco-induced oral mucosal lesions.

**Table 10:** Methylene blue, Lugol_Iodine and Epithelial dysplasia Crosstabulation.

		Epithelial dysplasia		Total
		Positive	Negative	
Methylene blue & Lugol_iodine	Positive	True positive = 19	False positive = 10	29
	Negative	False Negative = 0	True Negative = 21	10
Total		19	31	50

Percentages are based on a total sample size of *n* = 50.

**Table 11:** Diagnostic evaluation of Methylene blue and Lugol iodine versus Epithelial dysplasia.

Statistic	Value	95% CI
Sensitivity	100.00%	82.35% to 100.00%
Specificity	67.74%	48.63% to 83.32%
Positive Predictive Value (*)	65.52%	53.29% to 75.99%
Negative Predictive Value (*)	100.00%	83.89% to 100.00%
Accuracy (*)	80.00%	66.28% to 89.97%

(*) These values are dependent on disease prevalence. Percentages are based on a total sample size of *n* = 50.

### Behavioural response to vital staining

The mean score on the Contemplation Ladder following the staining procedure was 7.1 with a standard deviation of 2.7. The distribution of participants across the stages of change is presented in Table 12. A significant proportion of participants (72%) expressed a willingness to quit, with 21% planning to quit within the next 30 days. Qualitative feedback from patients often included expressions of concern and a heightened awareness of the health implications of their tobacco use, with many spontaneously requesting advice on cessation strategies.

**Table 12:** Motivation to quit tobacco following vital staining, as measured by the Contemplation Ladder (n = 50).

Total Percentage	Ladder of Change (Willing to Quit)
31.2%	I have made quit attempts in the past, but not successful
11.3%	I have made changes in my tobacco use, but I need to keep working at it
8.5%	I have begun to make changes in my tobacco use
9.6%	I plan to quit tobacco in the next 30 days
11.4%	I plan to quit tobacco in the next 6 months
**Total Percentage**	**Ladder of Change (Not willing to Quit)**
5.9%	I have never made quit attempts in the past
8.3%	I sometimes think about quitting, but I have no plans yet
7.4%	I rarely think about quitting, and I have no plans to quit
3.1%	I do not think about quitting tobacco
3.3%	I have decided to continue tobacco use

## Discussion

This study examined the diagnostic utility of sequential methylene blue (MB) and Lugol’s iodine (LI) vital staining for detecting epithelial dysplasia in oral potentially malignant disorders (OPMDs) among tobacco users, a high-risk group often overlooked in conventional screening methods. Our main finding was a 100% sensitivity with dual staining compared to 94.74% sensitivity with MB alone, which highlights enhanced dysplasia detection. However, the specificity dropped from 87.10% to 67.74%, indicating a diagnostic trade-off that merits discussion.

### Methylene blue: strengths and practical considerations

MB’s high sensitivity aligns with extensive research. A prospective study by Muthu et al. reported MB sensitivity above 90% with moderate specificity, reinforcing its role as an adjunctive test rather than a stand-alone diagnostic tool [18]. Other studies have reported MB sensitivity ranging from 89 to 92%, with specificity between 66 and 70% [6, 23]. These findings are consistent with our results: MB alone reliably highlighted the majority of dysplastic lesions, but the risk of false positives persisted, particularly in inflamed or keratinized tissue, where dye retention may occur without underlying pathology [15].

### Augmenting diagnostic accuracy by adding Lugol’s iodine

LI selectively stains glycogen-rich normal cells, sparing dysplastic epithelium [24]. By combining MB with LI, our sensitivity reached 100%, eliminating false negatives, a key requirement for effective screening strategies. Comparable protocols in esophageal screening demonstrated improved detection of squamous dysplasia using MB and LI. Zhu et al. (2005) reported nearly perfect diagnostic accuracy (100% sensitivity, 97.7% specificity) in esophageal lesions using endoscopic staining [25]. The pathophysiological similarities between oral and esophageal mucosa support extrapolating such success, though anatomical and lesion-type differences may influence real-world oral diagnostics [26].

### Comparative insights from toluidine blue and dual-stain studies

The diagnostic principle of combining a nuclear stain with LI mirrors other vital staining protocols, such as toluidine blue (TB). It has been performed to detect oral malignancy disease by Epstein et al. way back in 1992 and reported good diagnostic accuracy of dual staining [27]. An Indian study reported TB + LI achieving approximately 90% diagnostic accuracy, particularly in distinguishing inflammatory lesions from dysplasia or carcinoma [28].

### Dual staining: the sensitivity–specificity dichotomy

While our MB + LI protocol achieved perfect sensitivity, specificity dropped to 67.74%, a direct trade-off. Sharma et al. (2021) reported 100% sensitivity and specificity in a small leukoplakia cohort [29]. However, their homogeneous sample and limited lesion types likely reduced confounders. In contrast, our cohort included heterogeneous lesions like ulcerated, inflamed, keratotic lesions, which may influence LI retention and contribute to false positives [30]. The literature suggests that non-dysplastic conditions can trap dye due to mechanical or inflammatory properties rather than glycogen content, impacting specificity [31].

### Clinical and public health implications

Vital staining techniques like MB and LI are cost-effective, safe, and easy to deploy, ideal for low-resource community settings [1]. Warnakulasuriya and Kerr (2021) highlighted their potential for mass screening in resource-poor countries, enhancing early detection and reaching underserved populations [32]. Unlike advanced imaging modalities requiring infrastructure and training, vital stains are compatible with basic clinical environments, an advantage in regions with high oral cancer burden but limited access to specialist services [33].

### Behavioural impact: beyond the microscope

Our study also sought to evaluate the potential of vital staining as a behavioural motivator. The quantitative assessment using the Contemplation Ladder revealed a high level of readiness to quit among participants after visualizing their stained lesions, with a mean score of 7.1. This aligns with the concept of a ‘teachable moment,’ where a health event catalyzes behavioural change [34]. The vivid, objective evidence of mucosal change provided by the stains appears to bridge the gap between abstract risk perception and tangible personal health consequence, effectively initiating cessation conversations. While the cross-sectional nature of our study prevents us from reporting long-term cessation outcomes, a noted limitation, the significant increase in motivation to quit immediately following the procedure is a strong and well-established predictor of future quit attempts [19]. Future longitudinal studies are needed to directly link this intervention to sustained abstinence. A notable outcome of this study was participants’ emotional and behavioural response to stained lesions. Many expressed concern or surprise at the visibility of dysplasia, which prompted immediate tobacco cessation conversations. Zhu et al. (2005) reported similar findings in esophageal MB + LI staining, patients altered habits after visualizing mucosal changes [25]. Elimairi et al. (2017) affirmed that LI enhanced lesion visualization and patient engagement regarding oral health [31]. This intersection of diagnosis and behavioural intervention represents a promising yet underexplored strategy in public health [35, 36].

Strengths and Innovative Aspects

1. Real-world cohort: Recruiting habitual tobacco users with varied lesion types better reflects screening conditions in rural and urban Indian clinics, compared to narrowly selected cohorts.
2. Dual utility: Demonstrating both diagnostic potential and behavioural stimulus encourages broader adoption beyond pathology alone.
3. Rigorous histopathology: Correlating staining with biopsy ensures robust validation of diagnostic performance.

Limitations and Mitigation Strategies

- Sample size: The sample size is small with only 50 individuals, statistical power is limited. Larger, multicentre trials would yield more precise specificity estimates and subgroup analysis.
- Lesion heterogeneity: A variety of oral mucosal lesions were included of different aetiology including inflammatory, potentially malignant and malignant lesions, which could complicate the specificity. Having the same cohorts and conducting a study may have a better diagnostic performance.
- Mechanical dye retention: Non-dysplastic tissues may retain dye, especially under inflamed or ulcerated conditions. Surface irregularities favour mechanical retention of the dyes. Rinsing protocols and alternative pre-treatment agents may help reduce this confounding feature.
- Observer bias: Although clinicians were experienced, subjective interpretation remains. The use of standardised digital photography and artificial intelligence based assessment could standardize the interpretation of clinical images, like in digital radiography or cytology.
- Cross-sectional design: Without follow-up, we cannot assess whether early detection reduces morbidity or mortality. Longitudinal outcomes are essential.
- No comparison with advanced modalities: Adjunctive tools such as autofluorescence or narrow-band imaging were not evaluated; comparative studies would inform cost–benefit decisions in resource-poor settings.

Future Directions

1. Expanded cohorts with lesion subtypes: Large sample size with distinct cohorts of leukoplakia, erythroplakia, oral lichen planus, ulcerative lesions may be used to refine diagnostic metrics.
2. Multicentre longitudinal studies: Longitudinal studies with long-term follow-up at different centres would be good to standardize a vital staining protocol as a useful diagnostic adjunct for early detection of dysplasia in OPMDs and OSCC.
3. Digital adjuncts: Application of digital photography, image analysis, and machine learning to enhance diagnostic consistency [37].
4. Behavioural outcome research: Assessment of the fact if stained lesion feedback promotes sustained cessation of tobacco-related habits and improved oral health.
5. Integration with national programs: To evaluate dual staining incorporation into public health initiatives, such as India’s National Tobacco Control Programme [38, 39].

## Conclusion

Our study supports the dual staining protocol using sequential Methylene blue and Lugol’s iodine as a highly sensitive (100%) method for detecting epithelial dysplasia in OPMDs among high-risk tobacco users. While its specificity is reduced relative to MB alone, the method’s cost-efficiency, safety, and simplicity make it highly advantageous for large-scale screening in resource-limited contexts. Beyond diagnostic accuracy, vital staining provides immediate visual feedback and engages patients in cessation discussions, thereby proving to be a novel educational tool that resonates with preventative public health strategies.

By merging visual diagnostics with behaviour change potential, MB + LI staining aligns with the broader mission of early detection and tobacco cessation. However, optimal implementation requires acknowledgment of diagnostic limitations, commitments to confirmatory biopsy, and development of standardized digital tools. With further validation in larger, diverse patient populations and formal follow-up, this dual staining technique could emerge as a cornerstone in global oral cancer prevention.

## Data Availability

The datasets used and/or analyzed during the current study are available from the corresponding author on reasonable request.
